# A scoping review of family experience and need during end of life care in intensive care

**DOI:** 10.1002/nop2.14

**Published:** 2015-03-02

**Authors:** Maureen Coombs

**Affiliations:** ^1^ Graduate School of Nursing Midwifery and HealthVictoria University WellingtonWellington6242New Zealand; ^2^ Capital and Coast District Health BoardWellington Regional HospitalWellington6242New Zealand

**Keywords:** end of life, family experience, family need, intensive care, scoping review

## Abstract

**Aim:**

To scope systematically and collate qualitative studies on family experience and need during end of life care in intensive care, from the perspective of family members.

**Design:**

Scoping review of qualitative research.

**Methods:**

Standardized processes of study identification, data extraction and data synthesis were used. Multiple bibliographic databases were accessed during 2011 and updated in 2013.

**Results:**

From an initial 876 references, 16 studies were identified for inclusion. These were predominantly single site, North American studies that explored issues relating to the temporal stages in the end of life trajectory and the requirement for information and emotional support at end of life. With a strong focus on family need and experience during the transition from active treatment to end of life care, more work is required to understand how doctors and nurses can support families from treatment withdrawal through to death.

## Introduction

Internationally there has been increasing attention placed on improving end of life care as evidenced by the many international health initiatives (National Gold Standard Framework 2011, Kaiser Health 2013, New South Wales Ministry & Health 2013). Underpinning many of these policy drives is the concern for increased patient choice and a more collaborative approach involving the patient and healthcare providers when making decisions about care at end of life.

Over the past decade, there has been a similar growing interest to develop intensive care practices for those at the end of life. For societal perception of intensive care providing curative, life‐sustaining therapies, significant numbers of critically ill patients do not survive intensive care and die after receiving end of life care (Barber *et al*. 2006). International data demonstrate mortality rates of 20% in this setting with the majority of non‐survivors receiving end of life care through planned treatment withdrawal (Frick *et al*. 2003). As only small numbers of patients remain conscious during their critical illness (Wunsch *et al*. 2005), family members become the voice of the patient to inform decision‐making about goals of care (Kentish‐Barnes *et al*. 2009). Therefore, families are often central in decision‐making about end of life care in intensive care.

### Background

The role of the family is complex in intensive care. Informing care decisions is but one of the functions that families hold in this setting, with others including: caregiver, representing the patient's views by proxy and family spokesperson (Quinn *et al*. 2012). The diversity of such roles places significant demands on family members and this has been recognized by clinicians and academics alike. To date, literature on families in intensive care has focussed on assessing the generic needs of all families when visiting intensive care (e.g. Molter 1979) and on the development of interventions to generally improve communication with families (e.g. Scheunemann *et al*. 2011).

One of the important issues to emerge from this body of work is an increased understanding of the impact that intensive care has on families (Davidson 2009) and on the longer term health outcomes on this group, especially for those who are bereaved in intensive care. In one prospective longitudinal cohort study, Anderson *et al*. (2008) assessed anxiety and depression, post‐traumatic stress and complicated grief scores in 50 family members of intensive care patients (survivors and non‐survivors). Measurements were taken at enrolment, at 1 month and then 6 months. While the total sample demonstrated elevated levels of anxiety and depression, of the 38% who were bereaved, 46% (95% CI 22–71%) had complicated grief at the 6 month period. Important issues for family members appear to focus on how events were understood by families in intensive care and how families were supported at this time. There is, therefore, need to comprehend, from the perspective of families, what their experiences are during end of life care in intensive care and what their needs are at this time. This can then inform how care is delivered to best support family members who will experience bereavement in intensive care.

This paper reports on a scoping review of the literature on family experiences and need during end of life care in intensive care. This review was undertaken to inform development and design of a qualitative study in this area. As researching any bereaved population is a sensitive and potentially emotionally distressing activity for participants (Wiegand *et al*. 2008), it is important that a clearly identified knowledge gap be identified prior to interviewing this vulnerable group. Therefore, a scoping review, a systematic approach used to map the literature to identify areas empirically well‐explored while highlighting areas still to be explored (Ehrich *et al*. 2002), was seen as an important part of the research planning process. As this review was undertaken to inform development of qualitative research study in this area, only qualitative studies were included.

The scoping review question was: What is known in the qualitative research literature about the experiences and needs of family members during end of life care in adult intensive care from the perspective of bereaved family members?

For the purposes of the review, the following definitions were used:

- End of life care was defined as ‘the supportive and palliative care needs of both patient and family… identified and met throughout the last phase of life and into bereavement’ (National Council for Palliative Care 2006, p.2).
- Family member was defined as the partner, significant other(s) or relative of the person receiving intensive care. The term ‘family member’ was used due to the lack of consensus definition in the literature on the term ‘family’. Intensive care was defined as a clinical area providing ‘the monitoring and support of critically ill patients who have illnesses with the potential to endanger life’ (Valentin *et al*. 2011).

## The study

### Design

Scoping review of the qualitative literature.

### Methods

The method adopted for this scoping review was informed by Arksey and O'Malley's (2005) framework. Levac *et al*.'s (2010) recommendations for refining the scoping review methodology were further incorporated to increase rigour of the review process. Multiple electronic databases and key search engines were accessed using a specific search strategy. Records were identified using explicit inclusion/exclusion criteria and carefully formulated search terms. A standardized set of procedures for data extraction and data synthesis were developed and adhered to. Independent reviewing at study identification and data extraction stages occurred and was verified by another researcher. Standard to any scoping review, the analytical focus for this work was on critique of relevance, credibility and contribution of identified studies, rather than consideration of methodological strengths and weaknesses (Arksey & O'Malley 2005). Results were presented as descriptive numerics and textually.

### Search strategy and data sources

Literature searches were designed to retrieve papers from a range of academic disciplines via electronic databases including: CINAHL (Cumulative Index to Nursing and Allied Health Literature), Medline, EMBASE, Psychlit, PschINFO, Web of Science, Web of Knowledge. Other data sources, for example, Google Scholar were used to uncover additional material. Further electronic searches of major research registers were also undertaken including: National Institute for Health Research, Cochrane Library, together with high profile clinical academic centre websites with output in this area (University of Washington, Seattle and Joanna Briggs Institute, Melbourne). Manual searching of key international UK, Australasian and North American) critical care and palliative care journals together with review of international critical care organizations websites (American Association of Critical Care Nurses, British Association of Critical Care Nurses, European Critical Care Nurses Association, Australian Critical Care Nurses Association and New Zealand Nurses Organisation, Intensive Care Society and Intensive Care Societies Critical Care Patient Liaison Committee) was undertaken. The searches were conducted during 2011 and updated in 2013.

### Data selection

Studies were selected using specific inclusion and exclusion criteria (Table 1). These were applied to each database. As the intent of this scoping review was to develop an in‐depth qualitative understanding of the research area, only qualitative papers were included. Only papers published after 1995 were included as the first seminal study in this area was published in 1996. Furthermore, studies published prior to that date were unlikely to reflect current practice and research reporting rigour. Summary reports of untraceable studies were also excluded.

**Table 1:** Scoping review criteria

Inclusion criteria	Exclusion criteria
English language studies Papers published after 1995 Studies on adult patients/care settings Studies involving end of life care/bereavement All qualitative research: primary, secondary data	Non‐English language studies Papers published prior to 1995 Studies in neonatal and children care settings Studies involving brain stem death Biomedical data (e.g. drug trials, clinical trials) Quantitative research papers, opinion and commentary pieces, retrospective audit data review; individual patient case presentation

Key search words included: (MeSH heading) intensive care or critical care + ITU (abbreviation for Intensive Therapy Unit) or intensive care or critical care or ITU (keywords), (MeSH heading) family + relative or family member or carer or caregiver (keywords), (Mesh heading) experience + needs or need or coping or burden (keywords), (MeSH heading) death + bereavement + terminal care or end of life or death or dying (keywords). Boolean operators and SMART search facilities were used to further refine the searches. A subject librarian provided expert advice on the search terms and later reviewed the search strategy.

### Data extraction, analysis and synthesis

The references of all potential papers were read, duplicate references deleted and all remaining titles of papers reviewed for suitability. Any papers not immediately meeting the selection criteria, for example, primary research in the neonatal intensive care population were excluded from the review. The abstracts of remaining papers were retrieved and read for suitability against the inclusion criteria. Every fifth abstract underwent a further independent review by another researcher to ensure rigour and consistency in the review process. All remaining papers identified as suitable for full review were retrieved through web based or library resource. Bibliographic details, keywords and abstract of all suitable papers were imported into a bibliographic software package (Endnote). All final papers underwent two independent reviews by the author of this paper and another researcher. There was full agreement on all decisions regarding papers for inclusion and exclusion. Papers were read to identify study aims and purpose, methodology, analytical strategies and findings. Data extraction sheets were completed for each paper and presented as a summary of: author and year of publication; study population; purpose of study; methods including analytical approach; original study findings and commentary (including knowledge contribution and gaps: method and theory). Each data sheet was read and key areas were collated. Consistent with Arksey and O'Malley's (2005) approach, findings from each included paper were thematically analysed, with key themes developed pertinent to the scoping review question. To undertake this, an approach that drew on Braun and Clarke (2006) principles of thematic analysis was used. Reading and re‐reading of the study findings across the included papers achieved familiarization of the data. This led to the organization of findings across this corpus of work into meaningful groups and then finally into the key themes. Descriptive statistics on studies included and excluded in the scoping review were collated.

### Ethics

Ethics approval was not required.

## Results

**Figure 1:**
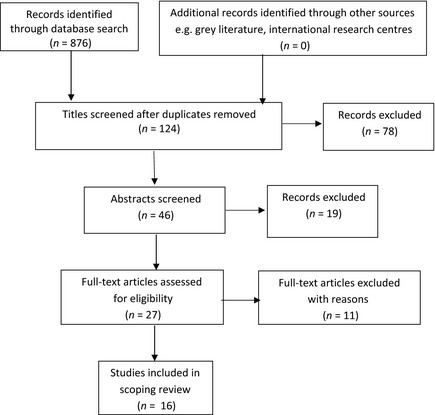
PRISMA flow diagram

The search strategy produced an initial 876 references (Figure 1). No new references were identified through other web‐based sources or manual searching. Duplicate references from across the different bibliographic sources (CiNAHL, MEDLINE, PsychINFO, PsycARTICLES) were discarded. Review of each paper's title revealed opinion and commentary papers, studies with a mixed paediatric and adult focus and papers incorporating aspects of brain stem death. Once these were excluded, 46 abstracts were retrieved and read. Of these, 27 full text versions were accessed via web based resource (*n* = 23) and via inter‐library loans (*n* = 4). Following critique of the full text papers, 16 were selected for this scoping review. Eleven papers were excluded at this stage as reading of the full text identified detail indicating that the papers did not meet the original inclusion criteria.

### Overview of scoping review literature

Prior to describing findings from this scoping review, an overview of the final papers will be presented. The majority of the review papers originated from the USA (*n* = 14). Other studies were from Canada (*n* = 1) and Sweden (*n* = 1). No papers were retrieved from other countries in Europe nor from Australasia. Predictably, most publications were located in specialized palliative care or critical care American journals (*n* = 14) with two papers being published in UK based journals (Table 2). As defined by the search criteria, all papers were published after 1996 with most being published after 2002. This speaks to the relatively recent interest and development of knowledge and practice in the area of end of life care in intensive care. Findings from the earlier seminal work (Swigart *et al*. 1996) appears to resonate with findings from recent studies. This suggests some constancy in the culture, practices and challenges of end of life care over the past two decades.

**Table 2:** End of life care scoping review – publication source

Publication source	No. of articles	Publication year(s)
American Journal of Critical Care	4	1998, 2002, 2003, 2006
American Journal of Hospital & Palliative Medicine	1	2009
Critical Care Medicine	1	2002
Critical Care Nursing Clinics of North America	1	2002
Critical Care Nursing Quarterly	2	2002, 2012
Heart and Lung	1	1996
Journal of General Internal Medicine	1	2012
Intensive and Critical Care Nursing	1	2009
Journal of Clinical Ethics	1	2005
Journal of Clinical Nursing	1	2008
Journal of Palliative Medicine	1	2008
Oncology Nursing Forum	1	2007

Thirteen papers gave accounts of primary research undertaken while three were secondary analysis (Table 3). In papers stating the research approach underpinning the study, five studies used phenomenology and three studies used grounded theory. One paper was a meta‐synthesis of the literature (Meeker & Jezewski 2008). This paper was included in the scoping review as it generated new knowledge and conceptualization of end of life care not previously explored. The research sampling strategy across the research papers was predominantly retrospective (*n* = 9) with two using prospective sample identification. Six papers used prospective sampling with a prospective data collection approach.

**Table 3:** Papers included in scoping review

Author(s)	Purpose	Study population	Methods	Key findings from original study
Swigart *et al*. (1996)	Describe the process of family decision‐making about life support.	30 family members of critically ill patients in one US ICU.	Descriptive prospective exploratory involving interviews, field notes and recorded family conferences.	Key inter‐related areas: understanding and reframing critical illness; reviewing and revising life story of the patient; maintaining family roles and relationships.
Jacob (1998)	Describe and explain family experiences when involved in decisions making in ICU.	17 adult family members involved in treatment withholding/limitation decisions in one US hospital.	Qualitative study using grounded theory method. Semi‐structured interviews occurring 1 week – 15 months into bereavement.	Major themes: Arriving at a judgement; moving in concert or disharmony; looking back and going on.
Abbott *et al*. (2001)	Identify psychological support and areas of conflict for families of ICU patients during treatment withdrawal/withhold in ICU.	48 family members in one US hospital.	Mixed method study. Sample identified prospectively but approach to relatives occurred 18–22 months after hospitalization. Descriptive demographic data and semi‐structured interview used.	46% of families reported family conflict in ICU. Health care staff perceived to be disrespectful of family members requests. 92% of families involved in decision‐making. Spiritual support important.
Counsell and Guin (2002)	Understand family needs during withdrawal of life support in the critically ill.	20 family members in medical ICU in one US hospital.	Descriptive design. Prospective sample identification. Telephone semi‐structured interview 3–5 weeks into bereavement.	Key needs: communication with family; access to quiet places; patient monitors to remain on during dying process; patient to look peaceful.
Kirchhoff *et al*. (2002)	Obtain detailed picture of the experiences of family members during the hospitalization and death of a loved one in the ICU.	8 family members from 8 ICUs in 2 US hospitals. Critically ill population under 55 years of age not included.	Qualitative design. Retrospective sample identification (6–18 months bereavement). Observed and taped focus groups included. Semi‐structured interview guide used. Content analysis used.	Key concept of experience as a vortex with issues of uncertainty; individual versus technological choice; responsibility to protect; staff and patient communication raised.
Warren (2002)	Explore critical care family members’ experiences of bereavement.	23 family members from one US ICU.	Heideggerian hermeneutic phenomenology. Semi‐structured interviews used. All participants bereaved within previous 12 months.	Helpful (e.g. information; unrestricted visiting) and unhelpful (e.g. Dr not available, not being present at death) experiences identified.
Norton *et al*. (2003)	Examine and describe communication difficulties from the perspectives of family members during withdrawal of life support.	20 family members from four tertiary care hospitals in the US.	Secondary analysis from larger study. Bereaved contacted 7–10 days after the death and interviewed 1–2 months later and then 6 months later. Semi‐structured interview used.	Unmet communication needs highlighted: give us information; just be straight with us; talk to us in lay terms; get us together as a team; listen to us.
Chamber‐Evans and Carnevale (2005)	Understand experiences of surrogates involved in making end of life decisions for a family member in an ICU.	8 surrogate decision makers in one Canadian hospital.	Prospective phenomenological study. Primary interview to gather data with second interview to check reliability.	Four themes: did I do the right thing; the struggle to set aside one's own convictions; the struggle to hold onto the whole story of the patient; maintain the dignity and identity of the patient.
McHale Wiegand (2006)	Describe interactions/experiences between family members, healthcare providers and the healthcare system.	19 families (56 family members) from 3 ICUs in a US hospital.	Secondary analysis from a larger interpretive phenomenological study involving prospective interviews with families. Descriptive phenomenology using van Manen's approach.	Key themes described: issues with health care providers (importance of relationships with staff, communication) and issues related to the hospital system (parking, privacy).
Limerick (2007)	Understand processes used by surrogate decision makers who have chosen to withhold and withdraw life‐sustaining measures in ICUs.	17 surrogates in across a Catholic managed multihospital (*n* = 4) US system.	Grounded theory design. Retrospective approach to surrogates. Semi‐structured interview guide used. Thematic analysis with axial coding.	3 domains used to represent decision‐making process: personal (e.g. rallying family support); ICU environment (e.g. developing relationships with staff); decision (arriving at new belief).
Meeker and Jezewski (2008)	Synthesize findings from investigations of family experience in decision to withdraw and/or withhold life‐sustaining treatment.	Metasynthesis of 13 qualitative studies described in 14 research reports and 3 dissertations published 1995–2007. Originated from US team.	Key search words included: life‐support care; withholding treatment; decision‐making. Intensive or critical care not used. Constant comparison technique and matrix mapping used.	Major categories: reframing reality involving cues and information; relating to care providers and family; integrating concerned with reconciling and going on.
Wiegand (2008)	Understand lived experience of families participating during withdrawal of life‐sustaining therapy in family member.	19 families (56 family members) from 3 ICU in a US hospital.	Secondary analysis from a larger interpretive phenomenological study involving prospective interviews with families. Descriptive phenomenology using van Manen's approach.	Main categories: this happens to other families; time to understand; time to see; rising a roller coaster; family readiness; willingness to consider; one step at a time; time to make a decision; family will go on; waiting for a miracle.
Fridh *et al*. (2009)	Explore close relatives’ experiences of caring when a loved one dies in an ICU.	17 close relatives of 15 adult patients who died in three ICUs in Sweden.	Phenomenological‐hermeneutic method. Open unstructured interview occurred 2·5–5 months after the death. Structural analysis undertaken.	Key themes: being confronted with loss; maintaining a vigil; trusting the care; adapting; facing death; need for privacy; experiencing reconciliation.
Radwany *et al. (* 2009)	Provide understanding of family experiences and emotional burden surrounding end of life decision‐making.	23 bereaved family members in one US ICU. All involved in a family conference led by palliative care services.	In‐depth semi‐structured interviews. Grounded theory guided data analysis.	Key temporal stages identified: the illness experience; decision‐making in the family meeting; the dying process. Three themes associated with emotional burden: lingering questions; resentment about care; feelings of guilt.
Gutierrez (2012)	Explore experiences and needs of family members for prognostic communication at end of life.	20 family members of patients in one ICU in a US hospital.	Prospective sample selection. Semi‐structured interviews with some follow‐up interviews. Content analysis used.	Five these of information‐related work: hearing and recalling; accessing; interpreting; retaining; utilizing information.
Schenker *et al*. (2012)	Characterize key intrapersonal tension experienced by surrogate decision makers in ICU.	30 surrogates from 5 ICUs at two US hospitals.	Prospective qualitative study. In‐depth semi‐structured interviews. Constant comparative technique used in analysis.	Intrapersonal tension around: responsibility for a loved one's death; chance of recovery; family well‐being. Five behaviours identified as coping mechanisms including sharing/delaying decision‐making and storytelling.

There was little consensus about definition of terms used in the studies that directed the research question and informed the sampling criteria. Life‐support, life‐sustaining, treatment limitation, treatment withdrawal and end of life were all used in the studies with little clarification offered. Similarly, although adult family members were the focus of all the studies, terms such as ‘surrogate decision makers’ and ‘loved ones’ were also used to describe the population. There was also variation across the age characteristics used to select the bereaved family member population; for example, one study excluded family members under 55 years of age.

With regard to data collection, most studies sampled at one time point with only two studies using longitudinal data collection. Two studies used statistical analysis mainly to describe demographic details of family members and to determine inter‐rater coding reliability during the analysis of interviews. Studies had sample sizes between 8–56 participants and most were conducted in single sites. Consistent with the interpretive paradigm used, most studies used recognized data analysis approaches, for example, constant comparison technique, axial coding. Rigour and ethics was variably detailed across the papers.

Finally, there was little detail of the underpinning theoretical frameworks that informed the research. There were examples of models generated in some of the studies and evidence of theory that underpinned some discussions. When such models and theories were used, more robust implications for future research and practice initiatives were often presented.

### Review of the experience and needs of families during end of life care in intensive care literature

The aim of this review was to scope and collate qualitative research that explored the experience and needs of families in end of life care in critical care. While some studies focussed on exploring family experience or family need, others were less defined in their approach. Across the 15 studies, nine were explicitly focussed on family experience at end of life, one paper focussed exclusively on family need during this time (Counsell & Guin 2002) and one paper focussed on family experience and need (Gutierrez 2012). Of the remaining studies, two studies (Abbott *et al*. 2001, Norton *et al*. 2003) focussed on families and communication difficulties/conflict at end of life and two studies (Swigart *et al*. 1996, Limerick 2007) on the processes used by families in end of life decision‐making.

However, the boundary between family need and family experience was unclear in most papers as there was often integration of family need and experience presented together in the reporting of the study findings (e.g. Meeker & Jezewski 2008, Fridh *et al*. 2009, Gutierrez 2012). Generally, studies that focussed on family experience described the emotional impact on families during end of life care (e.g. Wiegand 2008, Fridh *et al*. 2009). Studies focussing on family need reported on identifying communication and information requirements (e.g. Counsell & Guin 2002, Gutierrez 2012). While synthesizing findings from this scoping review, three key themes were prominent and related to: temporal stages of end of life in intensive care; information to make sense of end of life in intensive care; and emotional impact on families of end of life in intensive care.

### Temporal stages of end of life in intensive care

The end of life trajectory was described in most of the reviewed papers and mapped out through key stages that occurred over time. Wiegand's (2008) study exemplified this through using the chronology of treatment withdrawal process as part of the findings. In Radwany *et al*.'s (2009) work, the experiences of families in intensive care were likened to ‘a vortex’ with families entering the vortex, negotiating the vortex and finally leaving the vortex. Similarly, Jacob (1998) explored family members’ experiences with decision‐making in intensive care through the themes of ‘arriving at a judgment’, ‘moving in concert versus harmony’ and ‘looking back and going on’.

One of the key challenges associated with the end of life care in this clinical setting was the potential speed at which end of life care could proceed and the challenges this posed. In one study (Fridh *et al*. 2009) the length of stay from admission to time of death was 2 hours–5 weeks. From the findings reported across the papers in this scoping review, families needed information to cognitively process, understand and adjust to events leading to end of life care. Families also identified emotional support strategies that enabled adjustment to the experience of bereavement in intensive care.

### Information to make sense of end of life in intensive care

The importance of information to enable cognitive processing of events and make sense of end of life was a strong finding from this review. Key issues raised included the need for timely information (Norton *et al*. 2003) delivered in a consistent (Counsell & Guin 2002) and understandable format (McHale Wiegand 2006). Information from physicians and nurses was used by families to match against what families saw in critical care (Chamber‐Evans & Carnevale 2005) to come to terms with events.

However, detailed explanations of procedures and consequences was an area highlighted by families as lacking (Kirchhoff *et al*. 2002, Fridh *et al*. 2009). Comprehensive information was important as it acted as cues to families (Gutierrez 2012) and was significant in helping families understand events and reframe the critical illness (Swigart *et al*. 1996). Time was perceived an important factor to help families assimilate clinical information and come to terms with the severity of the illness and prognosis (Wiegand 2008). The emotional burden was highest for families who felt that insufficient opportunity had been given for questions or inadequate time had been given to make decisions (Radwany *et al*. 2009). If trust had been established with the clinical team, then information was perceived as holding more credibility (Swigart *et al*. 1996, Wiegand 2008).

Several studies highlighted that to process large amounts of information given in intensive care to family members, families identified the optimum environment for this to occur. Physical space was seen as important by families to create privacy for family discussion and decision‐making. Kirchhoff *et al*. (2002) and Fridh *et al*. (2009) identified the need for families to be near to, or present with the family member in intensive care. In addition, a range of resources were identified by families as being helpful at this time including: speakerphones in patients rooms to allow family members to communicate with staff, unrestricted visiting, a visitor's beeper, open visiting hours (Counsell & Guin 2002, Warren 2002) and flexible car parking practices (McHale Wiegand 2006).

### Emotional impact on families at end of life in intensive care

The emotional work undertaken by families was as a result of the intrapersonal and interpersonal turmoil experienced by families during end of life care. This was described by family participants in Wiegand's (2008) work as ‘riding a roller coaster’ and by Kirchhoff *et al*. (2002) as ‘a downward spiral of prognoses and difficult decisions leading to feelings of inadequacy and eventual loss’. Families expressed a need for reassurance that they had done the right thing in allowing treatment withdrawal for their family member and that they had been listened to as individuals and as a family unit (Norton *et al*. 2003, McHale Wiegand 2006). This helped families set aside their own beliefs and opinions about the confronting situation and actively engage in the decision‐making process. If physicians and nurses demonstrated respect when caring for the family member in intensive care, this too helped families when adjusting to end of life care events (Jacob 1998, Chamber‐Evans & Carnevale 2005). Families reported how retelling the family member's life story, at the bedside and in the private waiting room spaces, helped bring closure to the family member's life and make sense of the impending family member's death (Swigart *et al*. 1996, Limerick 2007).

Relationships with others were also important in providing emotional support to family members at this time. While relationships with wider family networks and with the clergy (Swigart *et al*. 1996, Abbott *et al*. 2001, Warren 2002) were all cited, it was relationships with health care providers that received greatest attention in this review. Indeed, a positive relationship with healthcare providers was perceived to help long‐term acceptance of the experience and decisions made in end of life care (Jacob 1998). The importance of this relationship was termed as ‘piloting’ the family through the end of life journey (Fridh *et al*. 2009). Supportive behaviours demonstrated by staff helped address the informational and emotional needs of families (Jacob 1998). When staff demonstrated respect for family members, this helped build relationships with family members (McHale Wiegand 2006) while unsupportive behaviours, for example, families pursuing medical staff for information, were perceived as destructive by family members (Limerick 2007). Families also identified that support was gained from coming together as a family unit (Limerick op cit) and helped build understanding that the family unit would continue after the death of the family member in intensive care (Wiegand 2008).

## Discussion

Results from this scoping review demonstrate a developing evidence base in qualitative understanding about family need and experience during end of life care in intensive care, albeit from a predominantly North American perspective. There are some well‐explored areas, for example, how information needs of families at the point of transition from cure to end of life are met. This work informs development of communication interventions (Lautrette *et al*. 2007) and tools to measure quality and satisfaction of care (Downey *et al*. 2010). However, other areas, such as when should information about transition to end of life be delivered and what language should be used to communicate this, have not received such in‐depth exploration.

Another area of note in this review was the role of the families in decision‐making at end of life. In earlier studies, there was little recognition of how families participated in decision‐making. This may reflect the culture or country of those early studies or result from the particular research lens brought to that work. However, given the increased importance placed on patient/consumer choice and involvement in care decisions and the different roles that families have in intensive care (McAdam *et al*. 2008), this must be an important area requiring further study in the future.

This scoping review also identified little theoretical underpinning in the reviewed studies. With bereavement theories well developed and used in other areas of health research (MacKinnon *et al*. 2014), bereavement theory could provide a lens with which to understand the experience of families in this care setting. It would be useful to explore application of, for example, of Stroebe and Schut's (1999) dual process theory of grief and the concepts of loss orientation and restoration orientation with the experience of bereaved family members in intensive care. Alternatively, research findings in the intensive care setting could be used to develop and test substantial bereavement theory. These are important considerations for future research.

As highlighted earlier, end of life care in intensive care can occur over a short period of time. This leads to challenges in conducting empirical work in matters of recruitment and data collection. Most studies in this review used retrospective sampling but a few studied participants over time to understand long‐term impact of bereavement. While undertaking qualitative end of life care research is challenging (Kendall *et al*. 2007) it is clear that there is need for exploration of long‐term impact on bereaved families in intensive care. Conducting research with vulnerable population such as bereaved families can be challenging for both researcher and those at the focus of the research.

As highlighted by Dickson‐Swift *et al*. (2007) researchers need to have awareness of the potential issues raised for participants in sensitive research and researchers need to be aware of the physical and psychological impact of collecting data about potentially difficult research areas. However, further such work would help identify whether bereaved family members are a homogenous group with regard to bereavement or whether certain attributes, for example, gender, nature of death (sudden, ‘expected’) predispose family members to higher risk of complex bereavement. Such information would help to develop targeted ICU bereavement interventions, an approach that has been advocated in other areas of bereavement support (Department of Health 2011).

While there was much discussion in the papers on meeting informational needs of bereaved family members, the importance of social relationships, outside of intensive care physicians and nurses, was less explored. The importance of existing family dynamics and social supports/networks and whether or how these foster family coping mechanisms in bereavement are yet to be explored.

Finally, this scoping review demonstrated a focus on the end of life trajectory in intensive care. This was often referred to as process with particular focus on the transition from cure to treatment withdrawal and the associated decision‐making. While the end of life trajectory has been explored elsewhere (Coombs *et al*. 2012), this scoping review demonstrated a lack of empirical study on the care given in the final hours of life, leading up to and after the point of death. The impact of care given at this time has not been specifically explored. This is an important omission in empirical work as this transition is often operationalized by nurses in intensive care (Long Sutehall *et al*. 2011). Understanding how family needs can be met at this time and how family experience can be improved is an important area and one that nurses can significantly impact on in practice.

### Strengths and limitations

As with any review, the comprehensiveness of the results rests with the review method chosen, in particular the search terms used. In this review, a previous metasynthesis on the experience of family decision makers in treatment withdrawal was identified (Meeker & Jezewski 2008) although, only seven of the sixteen papers were also cited by Meeker and Jezewski (op cit.) due to difference in the search terms used. Furthermore, the inclusion of quantitative studies in this review would have provided a more comprehensive review of the area but with limited contribution to informing the design of future qualitative research. The scope of this review was broad with a range of search terms used across the databases. No controls or restrictions were placed on how the search terms were defined across the data sources and this is a limitation. However, rigour of the search process was increased through use of a specialist librarian and use of a second researcher in the data selection, data extraction and data analysis stages.

A large proportion of this review literature originated in North America and its applicability in other countries cannot be assumed. However, similarity of findings across the review papers illustrate that family experience and needs at this time, may be similar across North America and Western Europe. Further work is required.

## Conclusion

Studies in this scoping review demonstrate the interest from practitioners and researchers to improve care for patients and families at the end of life. In this body of work on family need and experience in end of life care, there is broad consensus on key areas in this, for example, information, communication, relationships and support. However, this review has identified areas for further theoretical, empirical and practical exploration with the aim of improving family outcome in end of life care in critical care. Greater use of prospective and longitudinal studies is required to explore how time influences family perception of their experience and need. There is further opportunity to understand family need at the transition from treatment withdrawal to death in the intensive care setting and the impact of care at this time. As nurses manage care for patients and families at this pivotal time, this is an important area requiring further exploration and review.

## Conflict of interest

The author declares that there is no conflict of interest. This paper is based on work undertaken as part of a National Institute of Health Research & Department of Health Post‐doctoral Clinical Lectureship award, UK. It has not been published elsewhere.

## Author contributions

The author has solely contributed to the conception and design, acquisition of data, and analysis and interpretation of data; together with the drafting, writing and revision of the article.

All authors have agreed on the final version and meet at least one of the following criteria [recommended by the ICMJE (http://www.icmje.org/ethical_1author.html)]:

- substantial contributions to conception and design, acquisition of data, or analysis and interpretation of data;
- drafting the article or revising it critically for important intellectual content.
